# P05.12. The formation of scholars: doctoral education in the field of acupuncture and Asian medicine

**DOI:** 10.1186/1472-6882-12-S1-P372

**Published:** 2012-06-12

**Authors:** C Wilson

**Affiliations:** 1 American College of Traditional Chinese Medicine, San Francisco, USA

## Purpose

Practitioners of acupuncture and Asian medicine have begun to engage in doctoral education, however the impact of doctoral level education on this medicinal discipline is yet to be fully evaluated. This research examines three graduated cohorts and assesses the impact of doctoral education on clinical practice, employment, integrated health care settings and professionalism. This research surveys and interviews Asian Medicine practitioners that completed the doctoral program at the American College of Traditional Chinese Medicine (ACTCM) in San Franciso, CA. ACTCM began its first doctoral cohort in fall 2006. The 2.5 year Doctor of Acupuncture and Oriental Medicine (DAOM) program is offered 4 days a month (Friday through Monday), in an intensive format basis. It is a case-based program, focusing on practical and advanced knowledge and skills, collaboration with other health care practitioners, and scholarly activities. So far ACTCM has graduated three cohorts: 2009, 2010 and 2011 with a total of 36 alumni.

## Methods

Three graduated cohorts were surveyed and interviewed to assess and evaluate the impact that doctoral education has had on clinical practice, employment, integrated health care settings and professionalism. A mixed-methods approach was used to evaluate both qualitative and quantitative information.

## Results

This research project indicates that practitioners of acupuncture and Asian Medicine are entering doctoral programs for a variety of reasons associated with clinical practice and are interested in pursuing research in acupuncture and herbal medicine. Graduates that were surveyed indicated that there is value in doctoral level training and that this level of training is needed in order to move ahead in clinical practice, prepare to work in integrated healthcare settings, and to develop specialties in the field.

## Conclusion

Interest in postgraduate doctoral education is increasing.There is a growing interest in inter-professional medical education, integrated health care settings, and cross-disciplinary research.

